# Impact of Early Procedural Exposure on Pre-clinical Medical Students’ Confidence

**DOI:** 10.7759/cureus.77411

**Published:** 2025-01-14

**Authors:** Sonja Klumpp, Ingrid Rocha, Cyrus Amirfazli, Cameron Bear, Josh DeYoung, Mithil Gudi, Meet Patel, Daniel DeWeert

**Affiliations:** 1 Medicine, Wayne State University School of Medicine, Detroit, USA

**Keywords:** medical school curriculum, medical student competence, medical student confidence, procedural skills, simulation-based learning

## Abstract

Introduction: Pre-clinical involvement in procedural training is an essential part of medical school curricula and represents a core competency for medical students entering clerkships and residency. To prepare students for clerkships, "transition to clerkships" courses have been implemented just prior to entering the clinical setting. Despite this additional training, medical students often experience a lack of confidence in procedural skills. The purpose of this study is to determine whether expanding medical student exposure to procedural skills throughout the pre-clinical years improves confidence before entering a clerkship environment.

Methods: A cross-sectional study was performed at a large, single-campus medical school in which first- and second-year students attended an event introducing them to various procedural skills. Surveys were conducted to evaluate student perception of procedural exposure in their pre-clinical curriculum and the impact of the event on their level of confidence before entering clinical clerkships.

Results: One hundred eighty-seven medical students participated in the event and completed the surveys. Students reported a lack of procedural exposure in their pre-clinical curriculum and an increase in confidence to perform well in clerkships following the event. Statistically significant improvements in confidence were observed across all procedures taught during the event. Notably, volar wrist and thumb spica splinting showed one of the highest increases in confidence rating (mean difference: 5.686, p < 0.001), as did Foley catheter placement, for which students' confidence rating rose by 5.500 points (pre-event: 1.5; post-event: 7.0, p < 0.001).

Conclusion: Procedural exposure and training early in medical school can promote student confidence and success during clinical clerkships.

## Introduction

Despite opportunities to develop clinical skills in the pre-clinical years of medical school, students often enter their third-year clinical clerkships feeling unprepared and lacking confidence, which may result in suboptimal performance during clerkships. In a systematic review performed by Surmon et al., medical students entering clerkships were found to feel unprepared in several areas, including knowledge of their role on a care team and in performing transferable clinical skills such as those needed to participate in procedures [1]. This self-perceived lack of confidence can potentially hinder a student’s ability to become an active member of the patient care team and impede their development of interpersonal skills, which are both included in the Association of American Medical Colleges' (AAMC's) list of core competencies for entering medical students [2].

One area less commonly emphasized in the standard pre-clinical medical school curriculum is hands-on skills and procedural training [3]. For instance, there are many skills and procedures that students may be exposed to during their clinical rotations, including but not limited to suturing, phlebotomy, catheter placement, intubation, splinting, pap smears, lumbar punctures, and subcutaneous injections. Though third-year students may not be performing such procedures themselves, more robust pre-clerkship exposure to and greater knowledge of commonly performed procedures can encourage students to be more confident and seek out opportunities to become more involved in patient care. Valuable contributions may include explaining procedures to their patients, assisting in procedures, or completing simple yet helpful tasks such as retrieving necessary supplies.

This project aims to explore whether expanding medical students’ exposure to procedural skills through a one-day event during the pre-clinical years improves confidence before entering the clerkship environment. This was assessed through analysis of survey responses of students’ self-perceived confidence in various procedural skills before and after participation in a hands-on learning session. Results from this study can aid in future curriculum development by identifying and offering a strategy to help fill a gap in pre-clinical medical education.

## Materials and methods

This cross-sectional study took place at Wayne State University School of Medicine (WSUSOM), an urban, single-campus medical school in the Midwest. First- and second-year medical students voluntarily attended a one-day, three-hour event on August 17, 2023, designed to introduce them to various medical specialties via hands-on procedural skills. This event featured interactive, specialty-based workshops led by board-certified attending physicians with assistance from WSUSOM’s medical student interest groups. Specific procedures were selected based on their prevalence in hospital settings, feasibility of teaching within time constraints, suitability for classroom instruction, availability of teaching faculty, and cost of materials. Teaching and supervision were carried out by board-certified physicians, and proper safety protocols, including the use of sharps containers, were strictly followed.

Demographic data was collected for the 187 participants to verify their status as medical students and years in medical school. Pre- and post-event surveys were conducted to evaluate students' self-assessment of confidence levels, their perception of procedural exposure during the pre-clinical years, and the influence of the event on their confidence before entering clinical rotations. The post-event survey was administered immediately following the event. The surveys were administered through Qualtrics (Qualtrics International Inc., USA), and both pre- and post-event surveys consisted of six questions. Qualitative data were collected using a 10-point Likert scale (10 indicating high confidence).

To be included in the data analysis, students had to be in their first or second year of medical school, attend the entire event from start to finish, and have been assigned to three different procedurally based stations. The survey was offered to all students who met the inclusion criteria. Students were excluded if they did not complete both the pre- and post-event surveys in their entirety.

Data analysis involved paired t-tests to determine the significance between pre- and post-event participant responses. Standard deviations were also calculated, and a 95% confidence interval was used. The significance level was set at an alpha of 0.05. All statistical analysis was done using IBM SPSS Statistics for Windows, Version 29 (Released 2021; IBM Corp., Armonk, New York, United States).

## Results

A total of 241 medical students participated in the event, each exposed to three to six procedures from a selection of 16 different skills. However, only 187 students (78%) completed both pre- and post-event surveys (Table 1).

**Table 1:** Demographic class breakdown of participating medical students

Class	n	Percentage of participants
M1	148	79.03
M2	39	20.97

On average, the students' level of agreement that learning specialty procedures in the pre-clinical curriculum is important, and that learning these procedures will increase their confidence entering clinical rotations, was 8.7/10 and 8.9/10, respectively. However, their level of agreement that the medical school curriculum contains enough procedural education opportunities was only 4.4/10.

Statistically significant improvements in confidence were observed across all procedures taught during the event. Analysis showed a mean increase of 3.115 confidence scale points in knot tying (pre-event: 2.731; post-event: 5.846, p < 0.001), a 1.886-point increase in ECG lead placement and interpretation (pre-event: 2.543; post-event: 4.429, p < 0.001), and a 5.089-point increase in instrument suture tying (pre-event: 1.578; post-event: 6.667, p < 0.001). Other notable increases included volar wrist and thumb spica splinting, which showed one of the highest increases in confidence rating (mean difference: 5.686, p < 0.001), and Foley catheter placement, for which students' confidence rating rose by 5.500 points (pre-event: 1.5; post-event: 7.0, p < 0.001). Finally, the confidence rating in ultrasound-guided IV placement increased by 4.442 points (pre-event: 1.040; post-event: 5.481, p < 0.001), while airway intubation saw a 5.000-point increase (pre-event: 1.958; post-event: 6.957, p < 0.001).

**Figure 1:**
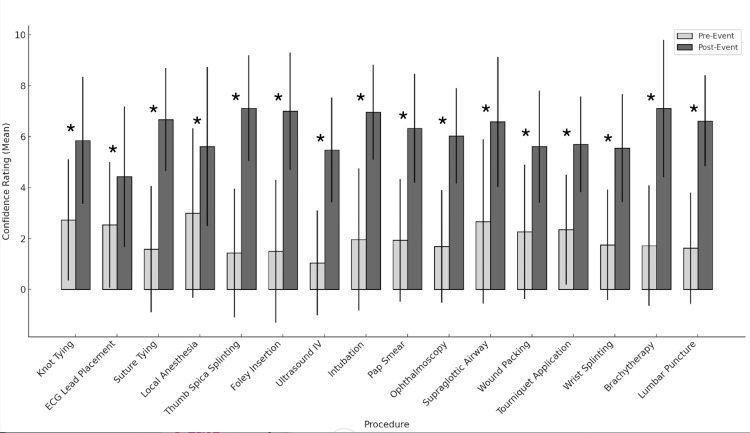
Comparison of pre-event and post-event confidence by procedure All comparisons marked with an asterisk (*) indicate a statistically significant difference of p < 0.05 using paired t-tests with pre-event confidence rating (10-point Likert scale) and post-event confidence rating (10-point Likert scale). A confidence rating of 10 indicates high confidence.

The detailed results of each procedure exposure, including the pre- and post-event confidence ratings, can be found in Figure 1 and Table 2.

**Table 2:** Comparison of mean confidence rating before and after teaching event by procedure type All comparisons were done using paired t-tests to compare the mean confidence rating pre-event and post-event for each participant. Ratings were done with a 10-point Likert scale (10 indicating high confidence), and significance was set at a level of p < 0.05.

		Pre-event confidence rating		Post-event confidence rating		Change in confidence rating (post-pre)		
Procedure	n	M	SD	M	SD	M	95% CI	p-value
Knot tying	26	2.73	2.38	5.85	2.49	3.12	2.10, 4.13	<0.001
ECG lead placement	35	2.54	2.47	4.43	2.76	1.89	1.12, 2.65	<0.001
Suture tying	45	1.58	2.48	6.67	2.02	5.09	4.33, 5.85	<0.001
Local anesthesia	18	3.00	3.33	5.61	3.13	2.61	0.96, 4.27	0.004
Thumb spica splinting	51	1.43	2.52	7.12	2.07	5.69	4.90, 6.47	<0.001
Foley insertion	52	1.50	2.80	7.00	2.31	5.50	4.66, 6.34	<0.001
Ultrasound IV	52	1.04	2.06	5.48	2.06	4.44	3.74, 5.14	<0.001
Intubation	23	1.96	2.79	6.96	1.87	5.00	3.82, 6.18	<0.001
Pap smear	43	1.93	2.41	6.33	2.14	4.40	3.72, 5.07	<0.001
Ophthalmoscopy	39	1.69	2.21	6.03	1.87	4.33	3.61, 5.06	<0.001
Supraglottic airway	12	2.67	3.23	6.58	2.55	3.92	1.90, 5.93	0.001
Wound packing	23	2.26	2.64	5.61	2.20	3.35	2.35, 4.35	<0.001
Tourniquet application	23	2.35	2.16	5.70	1.87	3.35	2.46, 4.24	<0.001
Wrist splinting	20	1.75	2.17	5.55	2.12	3.80	2.69, 4.91	<0.001
Brachytherapy	39	1.72	2.36	7.10	1.70	5.38	4.65, 6.12	<0.001
Lumbar puncture	45	1.62	2.19	6.62	1.78	5.00	4.33, 5.67	<0.001

## Discussion

This study explored whether exposing pre-clinical medical students to procedural skills impacted their confidence before entering clinical rotations, and our results confirmed that even a single event was enough to do so. In concordance with the literature, our results suggest that medical students believe there is not enough exposure to such skills in pre-clinical years. Thus, events such as the one carried out in this study are a good starting point for medical schools to promote confidence before students enter a hospital environment. While this study does not clarify the relationship between confidence and competence, the literature suggests that confidence alone can promote student learning and success. Additionally, with most medical school curriculums and USMLE Step 1 having transitioned to pass or fail, students need other ways of standing out; entering clerkships with greater exposure to procedural skills can provide an alternative way to do so.

Our results demonstrated that student participation in even a single-day, three-hour event was sufficient to augment their self-perceived confidence. Thus, we propose there is great value in students having even a single exposure over none, especially when the first exposure takes place in a low-stress environment. For instance, Battaglia et al. designed a training program in which medical students participated in simulation-based learning of procedural skills; their study suggested that simulation-based learning improved the anxiety and confidence of medical students and contributed to more effective clinical experiences [4]. Similarly, our study provides further evidence that being exposed to skills for the first time outside the hospital, where students can safely ask questions and make mistakes, is particularly useful for students to build confidence prior to entering the hospital environment. In addition, McKinley et al. demonstrated that exposure to skills during a pre-clinical boot camp was particularly impactful in helping mitigate student fears and enhance their perception of surgery before beginning their surgery rotation [5]. Therefore, early exposure to common procedures in various medical specialties also provides useful career exploration in addition to furthering student confidence. In addition, this single-day event was primarily organized by students with faculty oversight, utilizing borrowed supplies and the help of about 15-20 attending physician volunteers (in addition to residents), arguably only a mildly burdensome undertaking. Overall, our study suggests that exposure to skills even during a single-day event is impactful and a relatively easy addition to the standard medical school curriculum.

While our study does not equate confidence with competence, the value of confidence itself has been shown to be essential in promoting effective learning [6,7]. There is substantial anxiety associated with entering a clinical environment for the first time as a medical student, compounded by the expectation to navigate new and unfamiliar settings and interact with various medical professionals in a hierarchical system, all while being exposed to ill patients and emergency situations. Notably, research has shown that while confidence and competence are related, this relationship primarily emerges after students have undergone sufficient training. This underscores the importance of early procedural training sessions, which not only build important skills but also create opportunities to enhance confidence and therefore earlier development of competence [8].

Although it has been proposed that moderate stress can enhance learning and memory, excessive or chronic stress has been shown to have negative effects on learning [9]. This suggests that high levels of stress, discomfort, and unfamiliarity associated with entering the clinical environment may reduce the effectiveness of learning and procedural education. As such, increased confidence and reduced anxiety obtained through procedural skill training sessions provide a foundation upon which students will feel more comfortable in taking initiative during early clerkships and thus will gain more experience doing a variety of procedures they would otherwise not have felt comfortable engaging in [6,7].

Other studies have also shown that nursing students entering the clinical environment with higher levels of self-confidence after participating in simulation-based learning had greater confidence in performing tasks in the clinical environment [10]. Furthermore, in another study, medical students who reported higher levels of confidence received more teaching from residents and faculty during surgery clerkships, as their confidence signaled to instructors their readiness to engage in patient care [11]. This emphasizes the importance of cultivating confidence, not only for improved student performance but also to directly enhance their education.

With USMLE Step 1 transitioning to a pass-fail format, students now need additional ways to distinguish themselves on their residency applications. Much of the focus has shifted to how students perform in clerkships. In fact, a survey conducted by the National Resident Matching Program in 2021 found that 74.6% of residency program directors rank clerkship grades as a key factor when offering interviews [12]. While clerkship grading varies greatly among medical programs, it is generally calculated based on clinical performance evaluations, National Board of Medical Examiners (NBME) exams, and course assignments [13]. Increasing student confidence to perform well clinically is of particular importance, especially for introverted students who often have more difficulty in social environments such as hospitals and clinics. A study by the American Journal of Obstetrics and Gynecology investigating the impact of medical student personality type found a significant correlation between better clinical evaluations and extroversion [14]. For this reason, medical improv is a technique gaining traction within medication education aimed at assisting introverted students in clinical encounters by utilizing improvisational theater techniques. Literature has confirmed that medical improv aids in the development of competencies necessary to provide effective clinical care [15]. Events such as the one performed in our study may offer a similar experience by providing the opportunity for medical students to carry out a skill from start to finish as one would in a clinical setting. Therefore, these events can serve as another tool to assist students in overcoming barriers of introversion and excelling in their clinical performance.

There are limitations to this study that may affect the generalizability of the results. For one, the study focused on a single-campus event in which participating students were all exposed to the same pre-clinical curriculum. Curriculums at other medical schools may include varying levels of procedural training during the pre-clinical years, and events like the one analyzed in this study may have a different impact in the setting of a different curriculum. However, based on our knowledge and review of current literature, procedural training is a less emphasized part of the pre-clinical curriculum of medical schools at large, and results are likely still transferable. The study was also limited to students who voluntarily participated in the event, limiting the sample size and arguably targeting students who are especially motivated to pursue more procedural-heavy medical specialties. Another limitation is the use of a non-validated survey, and while the questions were inspired by those in validated surveys, they were developed to fit the specific event at the center of this study. The post-survey was also administered immediately following the event, which allowed time for a decrease in student confidence between the time of the event and the initiation of clerkships. Finally, while confidence is certainly a valuable quality for students to have before entering their clerkships, this study did not include long-term follow-up on the impact of this event during or after student participation in clerkships.

Overall, this study helps inform the advancement of present-day medical school curricula. Increasing the emphasis on procedural exposure and training, even through single-day large-scale events such as the one in this study, can enhance medical student confidence and readiness for entering a clerkship environment. As other studies have demonstrated, student confidence cannot be understated, as it can decrease levels of anxiety, enhance student learning, and help students stand out on the wards. Also, even a single exposure to a procedure is far more valuable than no exposure at all, suggesting that expanding upon procedural exposure during pre-clinical medical student training can be impactful without being burdensome or time-intensive. In addition, other studies have demonstrated the value of simulation and procedural training in the nursing curriculum, and these findings, as well as ours, can likely be expanded even further to encompass a broad range of healthcare professions. Because our study was limited by a lack of long-term follow-up, further investigation may include re-surveying students upon completion of their clinical clerkships. Additionally, our study informs another way in which students can stand out during clinical clerkships, especially those who are particularly introverted; future studies should expand on this, possibly by exploring the perspective of supervising residents and faculty regarding what procedures are most useful for students to be familiar with and other ways students stand out. Finally, there would be value in expanding our sample size to encompass students at various schools with different curricular opportunities beyond the standard medical school pre-clinical curriculum.

## Conclusions

In conclusion, our study results demonstrate the impact of early procedural exposure on pre-clinical medical students. Students showed notable increases in their level of confidence to enter clerkships after having exposure to various commonly performed procedures they will likely come across in a clinical setting. This study, in accordance with current literature, suggests that such exposure and learning are particularly beneficial when carried out in a simulated and low-stress environment. More broadly, early procedural exposure can set students up for success in their clerkships by furthering their ability to participate as active members of the care team and expanding learning opportunities. For these reasons, early procedural exposure, possibly through events such as the one carried out in this study, can be a valuable addition to standard medical school curricula.
